# Mixed-type autoimmune hemolytic anaemia presenting as multiple thromboses: A case report

**DOI:** 10.1016/j.amsu.2020.11.009

**Published:** 2020-11-06

**Authors:** Ganesh Kasinathan, Jameela Sathar

**Affiliations:** Department of Haematology, Ampang Hospital, Ampang, Selangor, Malaysia

**Keywords:** *Hemolytic anaemia*, *Thromboembolism*, *Direct antiglobulin test*, *Steroids*, *Direct oral anticoagulation*

## Abstract

Autoimmune hemolytic anaemia (AIHA) is a heterogenous disorder characterised by the presence of IgG or IgM pathological autoantibodies that target antigens of erythrocytes resulting in active hemolysis. Case presentation: A 40-year-old gentleman presented to a medical centre with chest pain and right sided hemiparesis for a week. He was pale and jaundiced. The power of the right upper and lower limbs was 3/5. His spleen was palpable. His complete blood count revealed macrocytic anaemia of 7.6 g/dL. The brain Magnetic Resonance Imaging (MRI) showed left fronto-parietal infarction. The right cardiac and left carotid angiogram revealed thromboses involving the right coronary and left internal carotid artery respectively. At the cardiology department, he was transfused with two units of red blood cells without his anemia being investigated and a stent was deployed to the left internal carotid artery. He was referred to the hematology department in which his peripheral blood smear revealed hemolysis and his direct antiglobulin test was positive. He responded to a course of steroids and direct oral anticoagulation and is in complete remission for the past 18 months. Conclusion: It is always imperative to investigate the cause of anaemia and consider hemolysis in a patient presenting with multiple unexplained thromboses.

## Highlights

• It is imperative to investigate anaemia and consider hemolysis in a patient presenting with multiple thromboses.
• Oral anticoagulation for three months would suffice for hemolysis induced thromboses.
• Unnecessary stent placement for provoked carotid artery thrombus results in long term anticoagulation.

## 1 Introduction

Autoimmune hemolytic anaemia (AIHA) is a heterogenous disorder characterised by the presence of IgG or IgM/C3d autoantibodies that target antigens of erythrocytes in warm or cold AIHA respectively, resulting in active hemolysis. The incidence of this rare disorder is estimated to be 0.8 per 100,000 [1]. AIHA is usually classified as warm, cold, or mixed in relation to the thermal range at which the pathological autoantibodies are most active. Microspherocytes are often a predominant feature on the peripheral blood film in warm AIHA while red blood cell agglutination is seen in cold AIHA. If features of anti-IgG and cold agglutinin titer of more than 1:64 are both not present, a diagnosis of paroxysmal cold hemoglobinuria (PCH) may be considered in the presence of anti-C3d and hypocomplementemia. A confirmatory test for PCH includes the Donath-Landsteiner analysis which demonstrates a biphasic haemolysin [2]. AIHA is often idiopathic while there are other causes that may contribute to secondary AIHA which include certain drugs, lymphoproliferative disorders, viral infections, and connective tissue diseases. Drug induced AIHA is usually characterised by the presence of anti-IgG on the direct antiglobulin test (DAT) with a negative elution reaction [3]. Viral infections such as Mycoplasma pneumoniae, Cytomegalovirus and Epstein-Barr, and lymphoproliferative disorders such as Waldenstrom macroglobulinemia are associated with cold AIHA. In cold AIHA, the anti-IgM/C3d is often detected on the monospecific DAT, with the antibody identification panel on room temperature showing panagglutination and elution studies being non-reactive [4]. Acute arterial and venous thromboembolism are widely known as potentially devastating complications of AIHA. There are many mechanisms which contribute to the prothrombotic state in AIHA. This case report aims to highlight the laboratory aspects of AIHA and the need for awareness among clinicians of such fatal consequences of acute thromboembolism in AIHA.

### 1.1 Case presentation

A 40-year-old Malay gentleman presented to a private medical centre with left sided chest pain associated with right sided hemiparesis and expressive dysphasia for a week. He had no past medical or family history. He was a married man with 2 children. He was a non-smoker and a teetotaller. He worked as a banker.

On examination, he was alert and pale with a tinge of jaundice. Proximal and distal pulsations were normal. The power of the right upper and lower limbs was 3/5 with brisk reflexes and upgoing plantar response. He had a palpable spleen of 2cm. Other systemic examinations were unremarkable.

His complete blood count revealed macrocytic anaemia of 7.6 g/dL with normal white cell and platelet parameters.

**Fig. 1:**
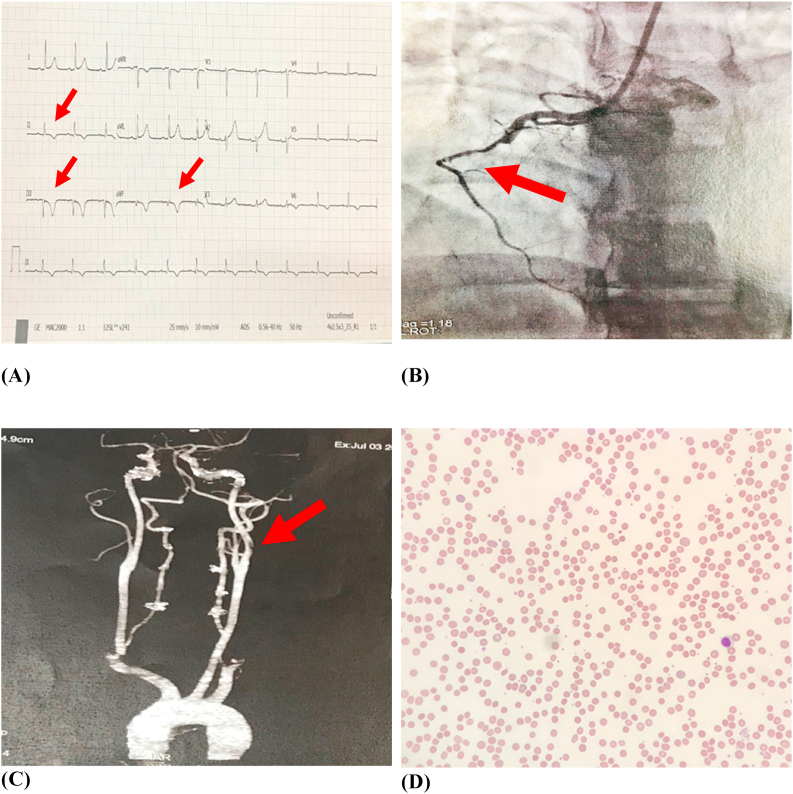
(A) 12 lead electrocardiogram showed deep symmetrical T wave inversion at the inferior leads. **(B)** Right sided cardiac angiogram showed occlusion at mid right coronary artery. **(C)** Carotid angiogram showed left internal carotid artery thrombus. **(D)** Peripheral blood film showed polychromasia, reticulocytosis and microspherocytes.

His 12-lead electrocardiogram (Fig. 1A) showed deep symmetrical T wave inversions at the inferior leads. The right sided coronary angiogram (Fig. 1B) depicted occlusion at the mid segment of the right coronary artery. The T2-weighted brain MRI portrayed left fronto-parietal infarction. A subsequent carotid angiogram (Fig. 1C) illustrated a 1.5 cm thrombus at the left internal carotid artery.

At the cardiology department, a carotid wallstent was deployed to the left internal carotid artery by the interventional cardiologist. He was transfused with two units of packed red blood cells without his anaemia being investigated further and was treated with dual antiplatelets for his Non-ST-Elevation Myocardial Infarction (NSTEMI).

He was referred to the hematology department for anaemia. The peripheral blood film (Fig. 1D) was consistent with hemolytic anaemia. The patient's ABO blood group using the automated method was A Rh (D) positive. The polyspecific and monospecific Direct Coombs Test (DCT) using antihuman globulin column agglutination card showed positivity in the presence of anti-IgG and anti-C3d autoantibodies. He had a low cold agglutination titer of 1:32 at 4 °C which was insignificant. Antibody screening using LISS (low ionic strength saline) and AHG (antihuman globulin) enhancement media showed panagglutination in cell I and II at 37 °C. The 11-cell antibody identification panel revealed panagglutination and a positive auto-control. Acid-elution studies with a commercial kit, in which, the eluate was tested against a panel of cells, demonstrated panreactivity. Last wash was negative validating the elution and wash process. The eluate was primarily IgG. The patient's RBCs were serologically phenotyped as R1R1, Jka + b-, Fya + b-, kk, NN, ss. Differential (allogeneic) adsorption was performed as there was a history of recent transfusion. No reaction was seen with donor R1R1, R2R2 and rr cells, thus confirming the presence of IgG autoantibodies. His antiphospholipid autoantibody and connective tissue screening tests were negative. The other laboratory parameters are tabulated in Table 1.

**Table 1:** Tabulation of laboratory parameters.

Laboratory parameters	Values (unit and normal range)
Hemoglobin	7.6 (13.5–16 g/dL)
Mean Corpuscular Volume	101 (80–95 fL)
Reticulocyte Count	7.5 (0.5–2.5%)
Direct Coombs Test	3+, IgG: 2+, C3d: 2+
Lactate Dehydrogenase (LDH)	560 (90–180 U/L)
Alanine Aminotransferase	38 (0–40 U/L)
Creatinine	90 (40–100 μmol/L)
Erythrocyte Sedimentation Rate (ESR)	80 (0–20 mm/hr)
Fasting Blood Sugar	4.4 (4.0–7.0 mmol/L)
Total Cholesterol	4.2 (<5.2 mmol/L)
Prothrombin Time (PT)	12.5 (9.5–13.5 sec)
Partial Thromboplastin Time (aPTT)	32 (27–38 sec)
Antinuclear Antigen (ANA)	Not detected
Lupus Anticoagulant (LA)	Not detected
Anticardiolipin antibody (aCL)	Not detected
Anti-beta-2-glycoprotein 1	Not detected
Troponin T	8.4 (<0.04)

He was diagnosed with mixed-type Autoimmune Haemolytic Anaemia with multiple organ thromboses (cardiovascular and neurological systems).

He was treated with oral prednisolone 1mg/kg daily for two weeks which was gradually tapered and is on long-term oral anticoagulation (rivaroxaban 20mg daily) for his left internal carotid artery stent. He has been in complete remission for the past 18 months.

## 2 Discussion

We describe an interesting case of a young male who had multiple thromboses due to active hemolysis without having other risk factors. The destruction of erythrocytes in mixed-type AIHA is intravascular and extravascular sequestration by phagocytosis in the spleen. The incidence of arterial and venous thrombotic manifestations is significantly greater in patients with AIHA and is frequently overlooked by treating clinicians. Our patient did not demonstrate any obvious risk factors.

Red blood cell (RBC) transfusion should be cautiously applied in asymptomatic patients with AIHA. Our patient was given red blood cell transfusion without the anemia being investigated. There is a risk of potentiation of hemolysis and RBC alloimmunization with injudicious use of RBC transfusion. Cross match compatible phenotype specific blood may reduce the risk of hemolysis due to alloantibodies being masked by an autoantibody but this does not eradicate the risk of hemolysis [5]. Furthermore, there are more than 400 erythrocyte antigens but our serological red cell phenotyping antisera may only a detect a known few and, the lifespan of transfused erythrocytes is shortened in AIHA [6]. The decision to transfuse should not be based merely on the haemoglobin level but on other important factors such as patient's clinical status, response to non-transfusion therapies and underlying co-morbidities such as heart or respiratory disease. However, in patients with cardiovascular compromise, RBC transfusion should not be delayed irrespective of haemoglobin level and transfusion in such cases generally remains safe.

Random case series have demonstrated that 10–25% of patients with AIHA who developed thromboembolism harboured antiphospholipid antibodies while 15% of AIHA patients who had thromboembolism did not have any antiphospholipid antibodies [7]. This leads to the fact that hemolysis itself contributes to thrombosis.There are various processes involved in the formation of arterial and venous thromboembolism in AIHA. Hemolysis and destruction of erythrocytes lead to abnormal exposure of phophatidylserine which contribute to the formation of tenase and prothrombinase complexes [8]. These complexes promote activation of coagulation pathways due to increased erythrocyte adhesion. Hemolysis of red blood cells also leads to nitric oxide depletion by free plasma haemoglobin further enhancing platelet aggregation and thrombus formation. Furthermore, thrombosis in AIHA is attributed to abnormalities in erythrocyte-endothelium interaction leading to elevated levels of tissue factor on vessel endothelium [9].

Thrombophilia testing should not be performed on patients who experience thromboembolism due to major risk factors as the outcome of the test will not change the indicated treatment of the patient [10]. American society of Hematology, via the Choosing Wisely campaign cautioned against injudicious practise of thrombophilia testing. False positive results will lead to inappropriate diagnosis of thrombophilia in patients and this results in unnecessary treatment without clinical need.

In our case, a carotid wallstent was deployed over the left internal carotid artery thrombus. A decision to place a stent was inappropriate in this case as it was not an internal carotid artery stenosis but a thrombus provoked by active hemolysis. Current indications for carotid artery stenting include symptomatic high surgical risk patients with more than 70% stenosis and asymptomatic patients with more than 60% stenosis [11]. Unnecessary usage of a stent requires long term anticoagulation due to the risk of acute in-stent thrombosis [12]. Ideally, oral anticoagulation for a duration of three months would suffice in this patient as it was a thrombus provoked by active hemolysis [13].

Thrombosis prevention by minimising acute episodic flares of hemolysis in AIHA is imperative to avoid lethal complications that may arise from thromboembolism. Glucocorticoids remain the first line therapy in idiopathic warm or mixed AIHA as seen in this case. High dose prednisolone at 1.0–1.5mg/kg per day for at least 2 weeks followed by a slow taper of 10 mg weekly over 8–12 weeks, with or without rituximab showed 50% of patients in the prednisolone-only-therapy group achieved complete or partial remission in 3 months [14]. Those who were treated with a combination of rituximab and prednisolone had higher complete remission rates at 12 months (75% versus 36%) [14]. Countries with restricted access to monoclonal antibody therapies or other second line agents may consider splenectomy as a viable second line treatment option with response rates of 60–90% [15]. Response rates are likely higher in patients without an underlying autoimmune disease. Other treatment options available for refractory AIHA are azathioprine, mycophenolate mofetil, danazol, cyclosporine and cyclophosphamide.

Prophylactic anticoagulation such as low molecular weight heparin should be given during active hemolysis in hospitalised patients until the hemolysis is stabilised [16]. Patients with coexisting antiphospholipid antibodies such as anticardiolipin and lupus anticoagulant are strong predictive risk factors for thromboembolism in AIHA and should benefit from prophylactic anticoagulation [17]. For AIHA patients with hemolysis-induced-thrombosis, a duration of oral anticoagulation of three months should suffice. Currently available oral anticoagulants include the vitamin K antagonist (warfarin) and the newer direct oral anticoagulants (DOAC). Warfarin requires close INR monitoring and, a time to therapeutic range (TTR) of more than 65% is desirable to ensure clinical efficacy. DOACs currently available include direct thrombin inhibitors such as dabigatran, and factor Xa inhibitors such as rivaroxaban, apixaban, edoxaban and betrixaban. Dabigatran requires concomitant use of a low molecular heparin for the first 5–10 days of therapy. Dabigatran is approximately 80% eliminated by the kidneys and may not be used in patients with a creatinine clearance of less than 30 ml/min [18]. On the other hand, rivaroxaban and apixaban demonstrate 30–80% and 25% clearance by the kidneys respectively and are usually contraindicated in patients with a creatinine clearance of less than 15ml/min [18].

DOACs have predictable anticoagulant effects and usually do not require routine monitoring. However, in certain situations such as haemorrhage, or in patients who require emergency surgery, measurement of the activity of DOACs may be desirable. Dabigatran activity is monitored using a dilute thrombin time (dTT) [19]. A normal thrombin time (TT) may exclude therapeutic plasma levels of dabigatran. Meanwhile, factor Xa inhibitor activity may be monitored using calibration-specific anti factor-Xa [19]. DOACs have demonstrated a lower risk of intracranial haemorrhage as compared to warfarin. In major or life-threatening haemorrhage, a monoclonal antibody known as idarucizumab given at a dose of 5g intravenously rapidly reverses the effect of dabigatran whereas andexanet alfa has been shown to reverse antifactor Xa activity in patients on rivaroxaban or apixaban [20].

## 3 Conclusion

It is imperative to investigate the cause of anaemia and consider hemolysis in a patient presenting with multiple unexplained thromboses. Oral anticoagulation for a duration of three months would suffice as it was a carotid artery thrombus provoked by active hemolysis and an unnecessary stent placement would subject him to inappropriate long term anticoagulation.

## Ethical approval

Ethical approval is not required as this is not a clinical trial.

## Consent

Written informed consent was obtained from the patient for publication of this case report and the accompanying images. A copy of the written consent is available for review by the Editor-in-Chief of this journal on request.

## Funding and sponsorship of this paper

Self-funding and no sponsorship received.

## Author's contribution

G.K. analysed the data, designed the paper and wrote the first draft of the manuscript. J.S. made critical revisions and approved the final manuscript.

## Guarantor

Ganesh Kasinathan is the guarantor of this manuscript.

## Provenance and peer review

Not commissioned, externally peer reviewed.

## Declaration of competing interest

The authors declare there are no conflicting interests.
