# Supplementary figures and images for: Mixed-type autoimmune hemolytic anaemia presenting as multiple thromboses: A case report
Source: Ann Med Surg (Lond). 2020 Nov 6;60:323–6. doi: 10.1016/j.amsu.2020.11.009 (PMC7653202; doi:10.1016/j.amsu.2020.11.009)

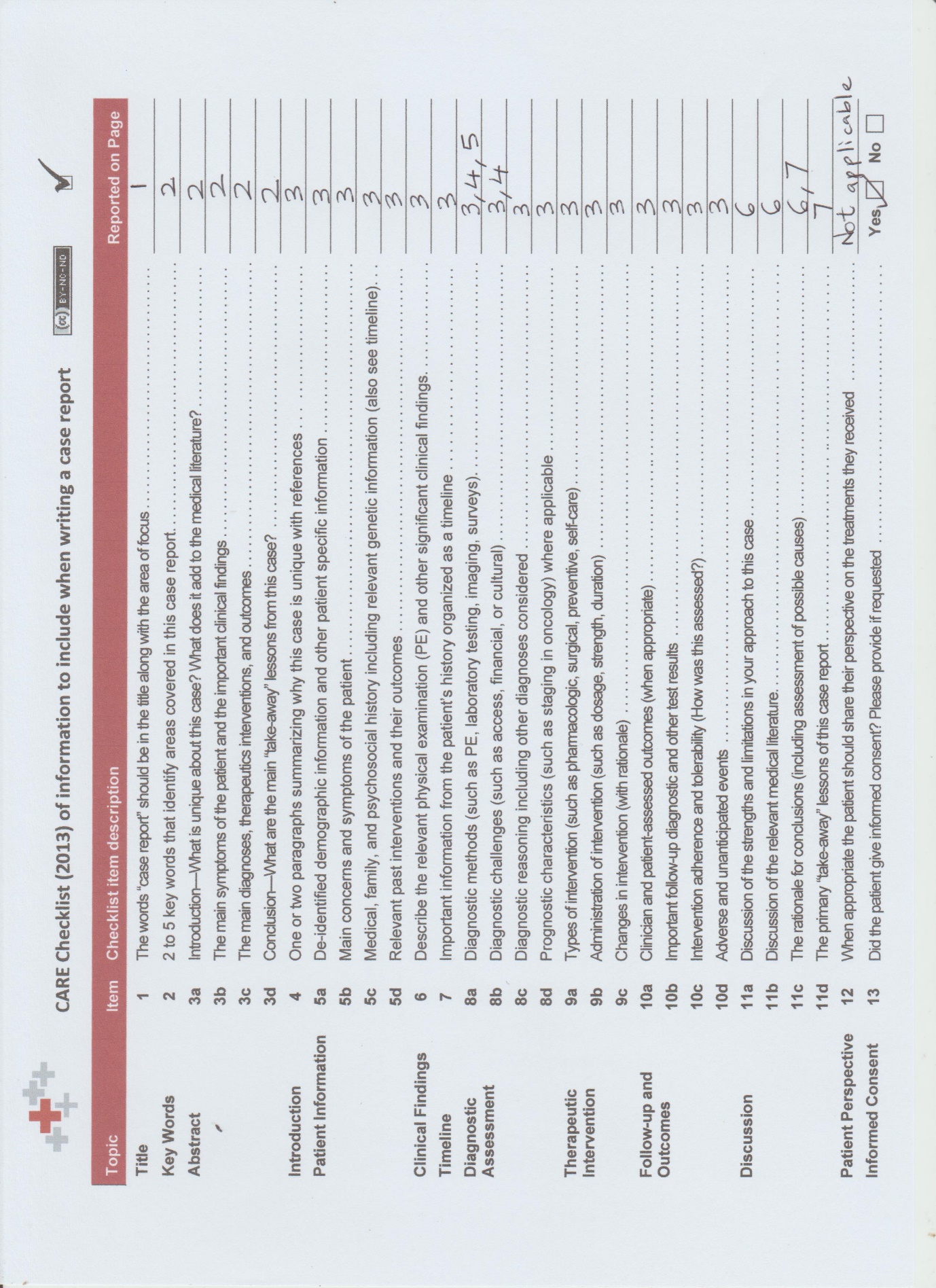

Supplement: Multimedia component 1 [file mmc1.docx]
